# Pantinin‐Derived Peptides against Veterinary Herpesviruses: Activity and Structural Characterization

**DOI:** 10.1002/cmdc.202500333

**Published:** 2025-09-14

**Authors:** Rosa Giugliano, Carla Zannella, Annalisa Chianese, Clementina Acconcia, Alessandra Monti, Roberta Della Marca, Ugo Pagnini, Serena Montagnaro, Nunzianna Doti, Carla Isernia, Massimiliano Galdiero, Filomena Fiorito, Luigi Russo, Valentina Iovane, Anna De Filippis

**Affiliations:** ^1^ Department of Experimental Medicine University of Campania "Luigi Vanvitelli" 80138 Naples Italy; ^2^ Department of Veterinary Medicine and Animal Production University of Naples Federico II 80137 Naples Italy; ^3^ Department of Environmental, Biological and Pharmaceutical Sciences and technologies University of Campania 81100 Caserta Italy; ^4^ Institute of Biostructures and Bioimaging (IBB) National Research Council (CNR) 80131 Naples Italy; ^5^ Department of Agricultural Science University of Naples Federico II 80055 Portici Italy

**Keywords:** antiviral peptides NMR analysis, scorpion venom peptides, veterinary herpesviruses

## Abstract

Animal viral infections represent a growing public health concern, as animals serve as reservoirs for pathogens, threatening food safety, biodiversity, and human health. In response, novel antiviral strategies are urgently needed. This study investigates the antiviral activity and structural properties of two antimicrobial peptides, pantinin‐1 and pantinin‐2, both derived from the venom of the scorpion *Pandinus imperator*, against caprine herpesvirus 1 (CpHV‐1) and bovine herpesvirus 1 (BoHV‐1). The results obtained from the plaque reduction assay and the quantitative real‐time polymerase chain reaction (PCR) indicate that synthetic pantinin‐mimetic peptides exhibited potent antiviral effects at concentrations ranging from 6–25 µM, impairing viral infectivity through direct virucidal action and inhibition of the viral entry and fusion with host cell. To characterize their structural behavior, nuclear magnetic resonance spectroscopy is performed in aqueous and membrane‐mimetic environments (trifluoroethanol (TFE)/H_2_O). In aqueous solution, both peptides predominantly adopted random coil conformations, with pantinin‐2 showing greater secondary structure propensity. In TFE/H_2_O, both peptides transitioned to *α*‐helical structures, which are often associated with membrane interaction and antiviral activity. These findings demonstrate that pantinin‐1 and pantinin‐2 possess promising antiviral properties, supporting their potential development as therapeutic agents against herpesviruses and other animal viral infections.

## Graphical abstract

Pantinin‐1 and pantinin‐2, scorpion venom–derived peptides, show potent antiviral activity against caprine and bovine herpesviruses. Acting via virucidal action and inhibition of viral entry/fusion, they adopt *α*‐helical structures in membrane‐mimetic environments. These structure–activity insights highlight their potential as therapeutic agents against herpesviruses and other animal viral infections.

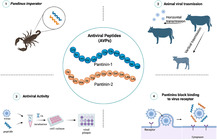

## 1 Introduction

Herpesviruses constitute a large family of enveloped double‐stranded DNA viruses classified into three subfamilies: *Alphaherpesvirinae, Betaherpesvirinae,* and *Gammaherpesvirinae*.^[^ ^1^ ^]^ Alphaherpesviruses are mainly characterized by a short replication cycle and the ability to invade neurons, establishing latency in sensory neurons.^[^ ^2^ ^]^ These viruses are critical human and veterinary medicine pathogens.^[^ ^3,^, ^4^ ^]^ Among the alphaherpesviruses infecting ruminants, bovine herpesvirus 1 (BoHV‐1) and caprine herpesvirus 1 (CpHV‐1) are of major concern due to their substantial economic and clinical impact.^[^ ^3^ ^]^ BoHV‐1 is a major etiological agent of infectious bovine rhinotracheitis (IBR) and is associated with a wide spectrum of clinical manifestations, including respiratory distress, nasal discharge, conjunctivitis, decreased milk production, pustular vulvovaginitis (IPV), balanoposthitis, infertility, abortions, and systemic infections.^[^ ^5,^, ^6^ ^]^ It also plays a central role in the bovine respiratory disease (BRD) complex, significantly compromising animal health and herd productivity.^[^ ^7^, ^8^, ^9^ ^]^ CpHV‐1, while antigenically related to BoHV‐1, primarily targets the genital tract in goats, leading to ulcerative vulvovaginitis, abortions, and high mortality in neonatal kids.^[^ ^10,^, ^11^ ^]^ The severity of infection is influenced by viral strain, host age, and the presence of co‐infections. Given its tropism and pathogenesis, CpHV‐1 is considered a biologically relevant model for studying genital herpesvirus infections and evaluating in vivo antiviral therapies, particularly those aimed at human herpes simplex virus type 2 (HSV‐2).^[^ ^1,^, ^12^ ^]^ Currently, therapeutic options against herpesviruses remain limited. Nucleoside analogs, such as acyclovir and cidofovir, are the pillars of treatment.^[^ ^13^ ^]^ For instance, cidofovir has been shown to relieve clinical symptoms and reduce viral shedding in goats experimentally infected with CpHV‐1.^[^ ^14^ ^]^ However, the utility of these antivirals is constrained by the frequent emergence of drug‐resistant viral strains, which underlines the need for new therapeutic approaches, in particular the identification/development of novel antimicrobial peptides (AMPs).^[^ ^15^ ^]^

AMPs are small, host‐derived peptides found across a wide range of organisms, including insects, amphibians, and mammals, and play a critical role in innate immunity. They exhibit broad‐spectrum antimicrobial properties, including antibacterial, antifungal, and antiviral activities, along with immunomodulatory effects,^[^ ^16^ ^]^ making them attractive candidates for the development of novel anti‐infective therapies. Mechanistically, AMPs typically disrupt microbial membranes through electrostatic interactions, leading to loss of membrane integrity and cell death.^[^ ^17,^, ^18^ ^]^ Additionally, many AMPs exert intracellular effects, affecting transcription, translation, or essential macromolecular structure synthesis, also causing the death of microbes.^[^ ^19^ ^]^

A subset of AMPs known as antiviral peptides (AVPs) possesses specific activity against viruses. These peptides are often cationic, amphipathic, and *α*‐helical in structure, and are derived from both plant and animal sources.^[^ ^20^ ^]^ Notably, peptides derived from scorpion venom have demonstrated potent antiviral effects by targeting multiple stages of the viral life cycle.^[^ ^21^, ^22^, ^23^ ^]^ The first peptide with virucidal activity identified from scorpion venom was Hp1090, which exhibited potent activity at low concentrations against the hepatitis C virus.^[^ ^24^ ^]^ Another scorpion venom‐derived peptide, BmKn2‐T5, demonstrated broad‐spectrum antiviral activity against several viruses, including enterovirus 71 (EV‐A71), Dengue virus (DENV), Zika virus (ZIKV), and herpes simplex virus type 1 (HSV‐1), particularly during the early stages of the viral replication cycle. Recently, the BotCl peptide with antiviral action against Newcastle disease virus (NDV) was isolated from the scorpion venom *Buthus occitanus tunetanus*.^[^ ^25^ ^]^

In this study, we investigate for the first time the antiviral properties of pantinin‐1 and pantinin‐2, two peptides isolated from the venom of *Pandinus imperator*,^[^ ^26^ ^]^ against BoHV‐1 and CpHV‐1. Pantinins belong to the non‐disulfide‐bridged peptide (NDBP) family, known for their multifunctional bioactivity.^[^ ^26^, ^27^, ^28^ ^]^ Although these peptides have shown notable antimicrobial effects, no antiviral activity has previously been reported. In this study, using synthetic peptides mimicking pantinin‐1 and pantinin‐2, we provide the first evidence that they act as effective AVPs. Furthermore, structural characterization of these peptides in aqueous and membrane‐mimicking environments offers valuable insights for the rational design of pantinin analogs with enhanced antiviral efficacy.

## 2 Results and Discussion

### 2.1 Dose‐Dependent Cytotoxicity Profile of Pantinin‐1 and Pantinin‐2 in MDBK Cells

**Figure 1:**
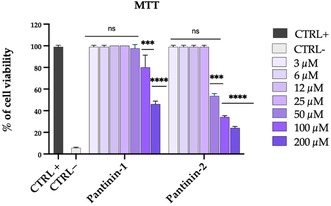
Cytotoxicity evaluation of pantinin‐1 and pantinin‐2 in MDBK cells using MTT assay. Untreated cells served as the positive control (CTRL+), whereas cells treated with 100% DMSO were used as the negative control (CTRL−). Cell viability was expressed as a percentage relative to the positive control. Data represent mean ± standard deviation (SD) of three independent experiments. Statistical analysis was performed using two‐way ANOVA followed by Dunnett's multiple comparisons test. Significance levels were determined in comparison to the positive control: ****p < 0.0001, ***p < 0.0002, ns = not significant.

The cytotoxicity of the synthetic venom‐derived peptides, pantinin‐1 and pantinin‐2, was assessed in MDBK cell lines using the MTT assay, which measures mitochondrial metabolic activity through the reduction of tetrazolium salt to formazan in viable cells. Results revealed a dose‐dependent cytotoxic effect for both peptides (**Figure** **1**).

Pantinin‐1 showed moderate cytotoxicity, with cell viability of 78% and 42% at concentrations of 100 and 200 μM, respectively. In contrast, pantinin‐2 exhibited a higher cytotoxic profile, reducing cell viability to 55% at 50 µM, 35% at 100 µM, and 25% at 200 µM. No significant cytotoxicity was observed at lower concentrations for either peptide.

The 50% cytotoxic concentration (CC_50_) was calculated at 113.5 for pantinin‐1 and 64 µM for pantinin‐2. These findings align with previous data by Crusca et al. who reported similar trends in fibroblast cells (HGF‐1), with pantinin‐1 displaying a CC_50_ of 97.5 and pantinin‐2 a CC_50_ of 43.5 μM, confirming the relatively lower cytotoxicity of pantinin‐1 compared to pantinin‐2.^[^ ^27^ ^]^ Based on these data, in subsequent assays, both peptides were tested in the concentration range 0 ÷ 50 µM, for a comparative analysis.

### 2.2 Antiviral Activity of Pantinin‐1 and Pantinin‐2 against CpHV‐1 and BoHV‐1

**Figure 2:**
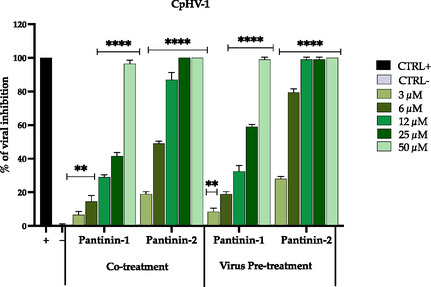
Antiviral activity of pantinin‐1 and pantinin‐2 against cpHV‐1 in cotreatment and virus pre‐treatment assays, expressed as a percentage of viral inhibition. The positive control consisted of cells treated with Oreoch‐1 peptide at 12.5 µg mL^−1^,^[^ ^54^ ^]^ while the negative controls included infected, untreated cells. Data represent three independent experiments’ mean ± standard deviation (SD). Statistical analysis was performed using two‐way ANOVA followed by Dunnett's multiple comparisons test. Significance levels refer to comparisons with the negative control: ****p < 0.0001, **p < 0.0021.

The antiviral potential of pantinin‐1 and pantinin‐2 against CpHV‐1 and BoHV‐1 was initially evaluated via a co‐treatment assay by quantifying plaque reduction within a concentration range of 3–50 µM. In this experimental setup, cells were simultaneously exposed to virus‐peptide mixtures at a 1:1 ratio. Both peptides exhibited a dose‐dependent inhibition of CpHV‐1 replication. Notably, we observed a complete viral inactivation observed at 50 µM for pantinin‐1 and 25 µM for pantinin‐2 (**Figure** **2**).

The half‐maximal inhibitory concentration (IC_50_) values were calculated at 30.4 and 6.1 µM for pantinin‐1 and pantinin‐2, respectively, indicating a markedly higher potency of pantinin‐2. Further enhancement of antiviral efficacy was observed in the virus pretreatment assay, wherein the viral inoculum was preincubated with the peptides before cell infection. Under these conditions, both peptides demonstrated increased antiviral activity, with pantinin‐2 achieving complete inhibition of viral infection at 12 µM. The corresponding IC_50_ values were reduced to 20.1 for pantinin‐1 and 4.3 µM for pantinin‐2, highlighting a more pronounced virucidal effect upon direct interaction with viral particles. These results strongly support the potential of pantinins, particularly pantinin‐2, as effective antiviral agents targeting enveloped DNA viruses.

**Figure 3:**
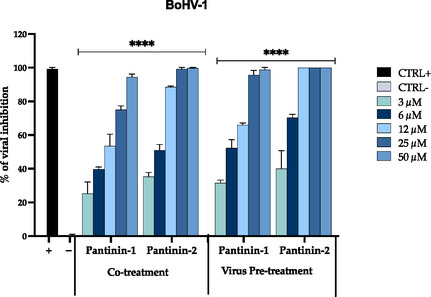
Antiviral activity of pantinin‐1 and pantinin‐2 against boHV‐1 in cotreatment and virus pre‐treatment assays. Positive and negative controls were represented by Oreoch‐1 peptide‐treated cells at 12.5 µg mL^−1^,^[^ ^54^ ^]^ and by infected and untreated cells, respectively. Two‐way ANOVA with Dunnett's test was used for multiple comparisons. Statistical analysis refers to the negative control, ****p < 0.0001.

To further assess the antiviral efficacy of pantinins, the same experimental protocols were applied to evaluate their activity against BoHV‐1. Results were similar to those obtained for CpHV‐1, with both peptides demonstrating potent inhibition of BoHV‐1 replication in co‐treatment and virus pre‐treatment assays (**Figure** **3**).

In detail, pantinin‐1 showed an IC_50_ of 10 in co‐treatment and 5.2 μM in virus pretreatment, while pantinin‐2 again displayed higher efficacy, with IC_50_ values of 6.6 and 3.9 μM, respectively. In contrast, no significant antiviral activity was observed in either cell pretreatment and post‐treatment conditions for both peptides, indicating that their mechanism of action likely involves direct interaction with viral particles rather than interference with cell surface or intracellular viral processes (data not shown). These findings are consistent with previous studies on scorpion‐derived peptides, such as Hp1090 from *Heterometrus petersii*, which exhibited a virucidal effect against Hepatitis C virus (HCV) by targeting early stages of infection (IC_50_ = 5 μM).^[^ ^29^ ^]^ Similarly, mucroporin‐M1 has been shown to inhibit a range of enveloped viruses, including measles, SARS‐CoV, and influenza H5N1, through a direct virion‐inactivating mechanism.^[^ ^30^ ^]^

### 2.3 Antiviral Activity of Pantinins during the Early Stages of Viral Infection

To gain deeper insight into the antiviral mechanism of pantinins, additional experiments were conducted to assess their activity during the initial stages of BoHV‐1 infection, specifically focusing on viral attachment and entry. Enveloped viruses, such as herpesviruses, initiate infection through a two‐step process involving: i) binding to specific receptors on the host cell surface and ii) fusion of the cellular and viral membranes, leading to the entry and the subsequent release of the viral genome into the host cytoplasm.^[^ ^31^ ^]^

**Figure 4:**
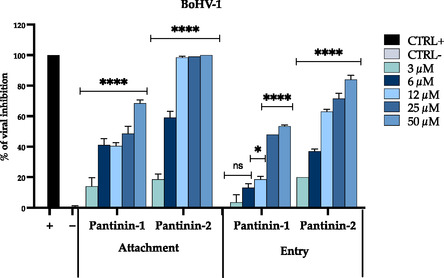
Antiviral activity in attachment and entry assay. Pantinins were able to block BoHV‐1 in the early phases of viral infection. Positive and negative controls were represented by treatment with Oreoch‐1 peptide at 12.5 µg mL^−1[^ ^54^ ^]^ and by infected and untreated cells. Two‐way ANOVA with Dunnett's test was used for multiple comparisons. Statistical analysis refers to the negative control, ****p < 0.0001, *p < 0.0332, ns not statistically significant.

As reported in **Figure** **4**, both pantinin‐1 and pantinin‐2 showed significant inhibitory activity against viral attachment, with pantinin‐2 displaying a greater efficacy (IC_50_ = 5.4 µM) compared to pantinin‐1 (IC_50_ = 26.2 µM).

To evaluate the impact of peptides on the subsequent membrane fusion step, a viral entry assay was conducted. Both peptides showed notable inhibitory effects on viral entry, with IC_50_ values of 39.8 for pantinin‐1 and 7.9 µM for pantinin‐2, further confirming their capacity to block fusion and prevent viral internalization. These results highlight the dual mechanism of action exhibited by pantinins in both the attachment and entry phases of the viral replication cycle. The greater antiviral efficacy of pantinin‐2 may depend on its greater propensity to form *α*‐helices compared to pantinin‐1. This aspect is already known to be important for interactions with the lipid bilayer of the viral envelope.^[^ ^32,^, ^33^ ^]^ The greater tendency of pantinin‐2 compared to pantinin‐1 to form *α*‐helices, already highlighted by previous circular dichroism experiments,^[^ ^34^ ^]^ was subsequently confirmed by our high‐resolution NMR structural analyses, as we will describe below.

Notably, similar mechanisms have been observed in other scorpion‐derived peptides. For instance, Kn2−7, an alpha‐helical peptide composed of 13 amino acids, was modified through the substitution of some amino acids, which led to an increase in the positive charge of the helix and greater anti‐human immunodeficiency virus (HIV) activity.^[^ ^35^ ^]^ The authors suggest that its structure and positive charge enable it to bind and integrate into the viral envelope.^[^ ^36^ ^]^ Li et al. showed that by increasing the alpha‐helix percentage of Mucoporin, there is an improvement in the antiviral activity.^[^ ^30^ ^]^ The peptide Eval418 and its derivative Eval418‐FH5, isolated from the scorpion *Euscorpiops validus*, were shown to interfere with HSV‐1 infection during the attachment phase, exhibiting IC50 values of 3.70 and 0.86 μg ml^−1^, respectively.^[^ ^37^ ^]^ These peptides, like pantinins, are short, cationic, and amphipathic, features that are known to promote strong electrostatic interactions with negatively charged viral membranes.^[^ ^38^ ^]^ The positive charge of pantinin‐1 and pantinin‐2 facilitates direct binding to the viral envelope, neutralizing surface charges and potentially disrupting membrane integrity. This interaction may result in the physical disintegration of the viral lipid bilayer, thereby inhibiting the ability of virus to proceed through the fusion and entry stages.

Taken together, these findings provide strong evidence that pantinin‐1 and pantinin‐2 target the early phases of viral infection, particularly attachment and membrane fusion, through a virucidal mechanism likely mediated by structural and electrostatic interactions with the viral envelope. These data are also confirmed by the absence of activity in pre‐treatment or post‐treatment assays (data not shown).

### 2.4 Cell Morphology Examination after Treatment with Pantinins

**Figure 5:**
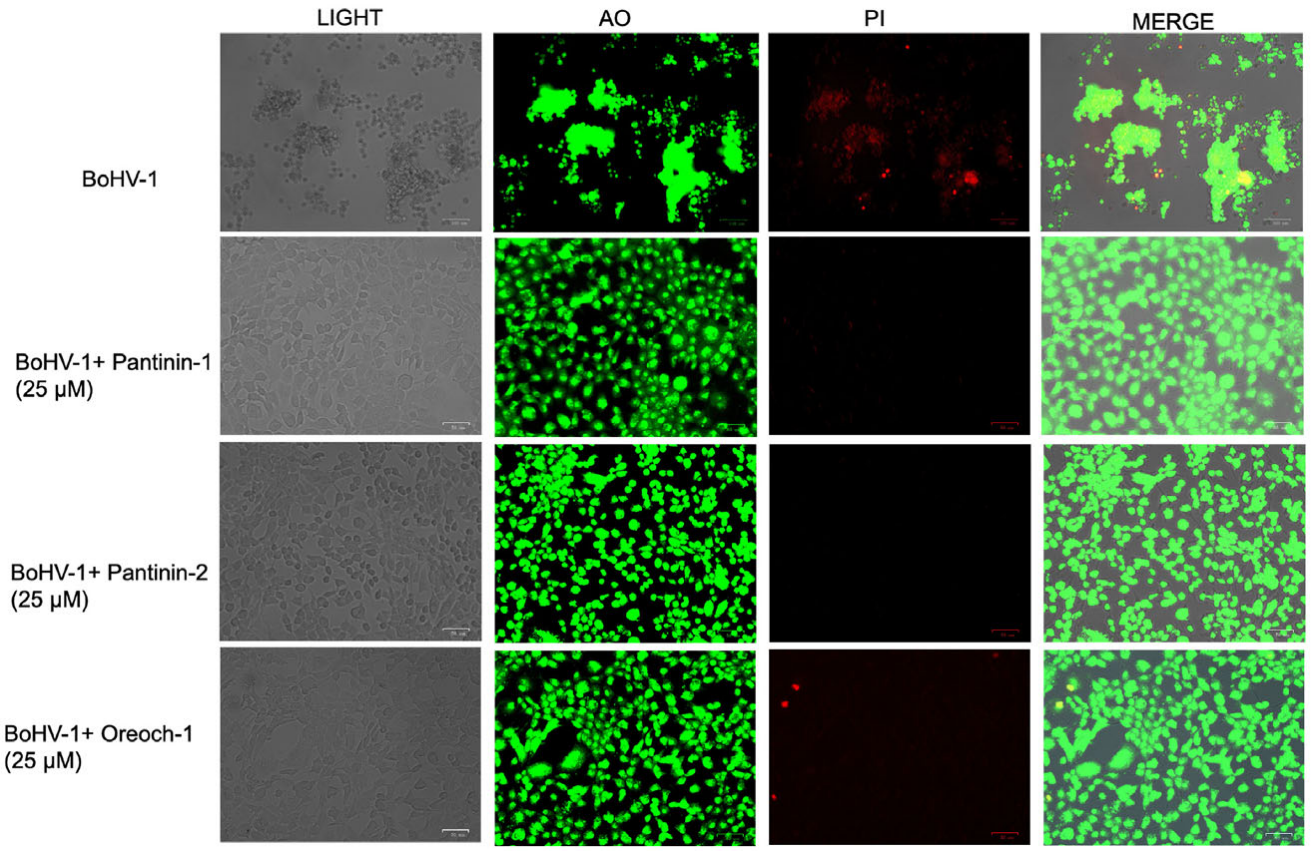
AO/PI fluorescence staining of MDBK cells following boHV‐1 infection and treatment with pantinins. Infected cells exhibited intense red fluorescence due to PI uptake, indicative of membrane‐damaged, non‐viable cells. Treatment with pantinin‐1 or pantinin‐2 (25 µM) markedly reduced or eliminated PI staining, with cells predominantly displaying green fluorescence from AO, consistent with preserved membrane integrity and viability. As positive control, Oreoch‐1 at 25 µM was used.

To further investigate the protective action of pantinins against virus‐induced cytopathic effects, a virus pre‐treatment assay was performed. Peptides and BoHV‐1 virions were pre‐incubated for 1 h to allow direct interaction, after which the mixture was dispensed to MDBK cell monolayers. Following 48 h of incubation, cell viability and morphological alterations were analyzed using acridine orange/propidium iodide (AO/PI) staining, a widely accepted technique for distinguishing live and dead cells based on membrane integrity (**Figure** **5**).^[^ ^39^ ^]^ AO, a nucleic acid‐binding dye, penetrates intact cell membranes generating a green signal upon binding DNA, marking viable cells. In contrast, PI selectively permeates damaged membranes, intercalates with DNA, emitting a red signal, which is indicative of non‐viable or membrane‐damaged cells.

Cells infected with BoHV‐1 displayed a pronounced red fluorescence; however, in cultures treated with pantinin‐1 or pantinin‐2, a marked reduction or complete absence of red‐stained cells was observed. Indeed, a predominance of green fluorescence was detected, indicating a significant preservation of cell viability in the presence of the peptides.

These findings suggest that pantinins effectively reduce BoHV‐1‐induced cytopathic effects: the increased number of viable cells in treated samples highlights the potential of these peptides to provide functional protection against herpesvirus infection by preserving cell membrane integrity.

### 2.5 Gene Expression Reduction after Treatment with Pantinins

**Figure 6:**
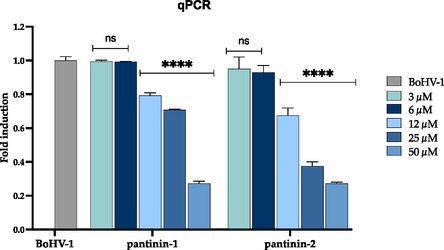
Quantitative real‐time PCR analysis of UL27 gene expression following virus pre‐treatment with pantinin‐1 and pantinin‐2. BoHV‐1 was pre‐incubated with peptides at different concentrations and applied to MDBK cells. Total RNA was collected after 30 h post‐infection, reverse‐transcribed into cDNA, and amplified. Infected cells not treated with peptides represent the positive control (see BoHV‐1 in the graph). Data are presented as mean ± SD from at least three independent experiments. Two‐way ANOVA with Dunnett's test was used for multiple comparisons. Statistical analysis refers to the positive control, ****p < 0.0001, ns not statistically significant.

Several viral glycoproteins participate in the attachment and entry of BoHV‐1 into host cells through a complex and multistep mechanism.^[^ ^3^ ^]^ Among them, glycoprotein B (gB), encoded by the UL27 gene, plays a pivotal role in membrane fusion during viral entry.^[^ ^40^ ^]^ Therefore, to assess the virucidal impact of pantinins, the transcription levels of UL27 were assessed by quantitative real‐time polymerase chain reaction (PCR) analysis, on infected MDBK cells, untreated and treated with peptides.^[^ ^41^ ^]^ Notably, both pantinins, at 50 µM, significantly reduced UL27 transcript levels, indicating a strong interference with the viral infection. Notably, the inhibitory effect provided by both synthetic peptides was dose‐dependent: as peptide concentrations decreased, UL27 expression progressively increased, ultimately approaching levels observed in untreated virus‐infected controls at 3 µM (**Figure** **6**).

These findings further support the virucidal potential of pantinins, and in particular of pantinin‐2, and highlight their capacity to disrupt key molecular events essential for BoHV‐1 infectivity.

### 2.6 Structural Features of Pantinin‐1 and Pantinin‐2 Probed by NMR

**Figure 7:**
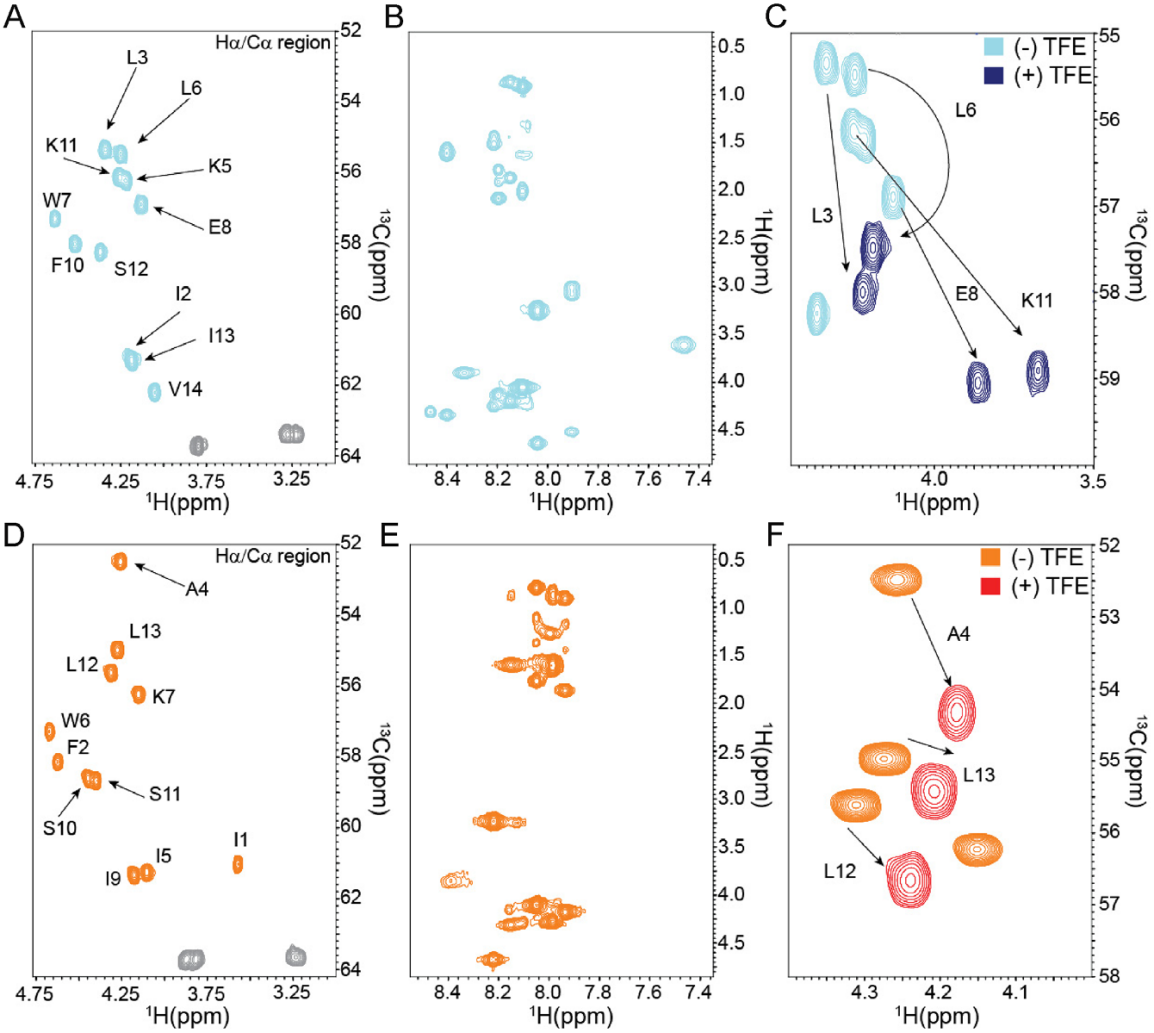
NMR measurements of pantinin‐derived peptides. H*α*/C*α* region of 2D [^1^H‐^13^C] HSQC, a portion of 2D [^1^H‐^1^H] TOCSY and 2D [^1^H‐^13^C] HSQC spectra acquired in aqueous solution for pantinin‐1 A–C) and pantinin‐2 D–F). In panels C and F, the 2D [^1^H‐^13^C] HSQC acquired in the presence of 30% TFE(v/v) is also reported.

To deeply explore the conformational properties of pantinin‐1 and pantinin‐2, we accomplished an accurate NMR structural investigation of the two peptides in an aqueous solution and membrane‐mimicking environment using the TFE/H_2_O mixture.^[^ ^42^ ^]^ A complete chemical shift assignment is a fundamental prerequisite to perform a high‐resolution NMR description of the structural peculiarities of peptides. This aspect is essential for peptides lacking ordered secondary structure elements, which in solution sample a highly heterogeneous conformational space. A nearly complete assignment of ^1^H, ^15^N, and ^13^C chemical shifts was obtained for the two pantinin‐derived peptides in the absence and the presence of TFE by applying a previously reported natural‐abundance NMR‐based strategy^[^ ^43^ ^]^ (Tables SI 1–8, Supporting Information). In details, we simultaneously analyzed homonuclear 2D [^1^H‐^1^H] TOCSY and 2D[^1^H‐^1^H] ROESY with 2D [^1^H‐^15^N] HSQC and 2D [^1^H‐^13^C] HSQC acquired by exploring natural isotopic abundance for the two peptides (**Figure** **7**).

**Figure 8:**
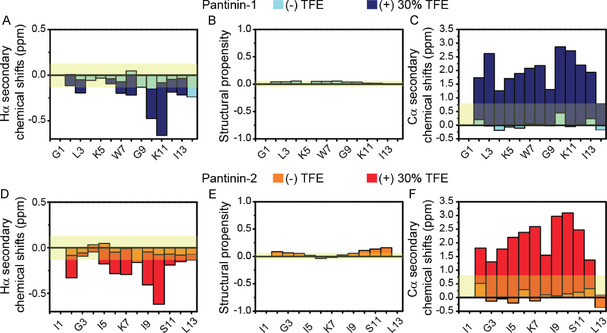
NMR‐based secondary structure analysis of pantinin‐derived peptides. H*α* secondary chemical shifts, secondary structural propensity (as revealed by neighbor corrected structural propensity), and C*α* secondary chemical shifts of pantinin‐1 A–C) and pantinin‐2 D–F) in aqueous buffer and the presence of 30% TFE. The light‐yellow rectangles in H*α* and C*α* secondary chemical shift plots indicate the cut‐off values for identification of secondary structure elements as proposed by Wishart and Marsh^[^ ^67,^, ^68^ ^]^ whereas the light‐yellow box in the structural propensity plots reports the threshold values allowing the identification of residues having significant secondary structure tendency.

Then, to capture the conformational characteristics of the pantinin‐derived peptides, we examined the backbone chemical shifts that are sensitive reporters of both structural and dynamical properties of the polypeptide chain.^[^ ^44,^, ^45^ ^]^ Moreover, the chemical shifts, like other NMR observables, are a population‐weighted average of the conformations adopted by the peptide in solution, and, therefore, they are a powerful tool for detecting transient secondary structures. In fact, while *α*‐helices and *β*‐strands are characterized by well‐defined chemical shift values, significant deviations of the H*α*, C*α*F4EA; C*β* and *H*_*N*_ shifts from the random or statistical coil values provide precise estimates of secondary structure populations.^[^ ^46,^, ^47^ ^]^ Therefore, to identify and quantify secondary structure elements, we analyzed H*α* and C*α* secondary chemical shifts (Δ*δ*) defined as the difference between the observed chemical shift (*δ*_obs_) and the per‐residue random coil (*δ*r.coil) value. The statistical coil values were predicted by using two different approaches as defined by Kjaergaard et al.^[^ ^48,^, ^49^ ^]^ and Tamiola et al.^[^ ^50^ ^]^ respectively. In addition, we also evaluated the per‐residue Δ*δ*C*α*‐Δ*δ*C*β* differences that represent a common procedure of reporting secondary structure. First, we analyzed H*α*, C*α,* and C*β* chemical shifts assigned for the pantinin‐1 and pantinin‐2 in an aqueous buffer. As shown in **Figure** **8**, for the two peptides, in the absence of the organic solvent, H*α* and C*α* slightly deviate from the corresponding random coil value indicating, in agreement with Δ*δ*C*α*‐Δ*δ*C*β* values (Figure S1, Supporting Information)F4EA; that the peptides are disordered and have no conformational preferences for well‐folded forms.

Interestingly, although the two peptides show similar conformational features, an accurate analysis of neighbor corrected structural propensity values indicates that pantinin‐2, in the C‐terminal region from Ile9 to Leu12, contains a level of transient helical secondary structure higher than pantinin‐1 (Figure 8). Then, we evaluated the backbone shifts observed for both peptides upon addition of TFE at a final concentration of 30% (*v/v*). According to previous CD studies,^[^ ^27^ ^]^ H*α* and C*α* secondary chemical shifts, as well as per‐residue Δ*δ*C*α*‐Δ*δ*C*β* values (Figure S1, Supporting Information)F4EA; clearly indicate that, in the presence of TFE, the two peptides adopt a stable helical structure encompassing the region from the residue located along the peptide sequence in the second position (i.e., Ile for pantainin‐1 and Phe for pantainin‐2) to the residue closed to the C‐terminal end (i.e., Val14 for pantainin‐1 and Leu12 for pantainin‐2). Overall, NMR data demonstrates that the pantinins, despite having a comparable primary sequence, show different conformational proprieties.

### 2.7 3D Structural Models of Pantinin‐Derived Peptides

**Figure 9:**
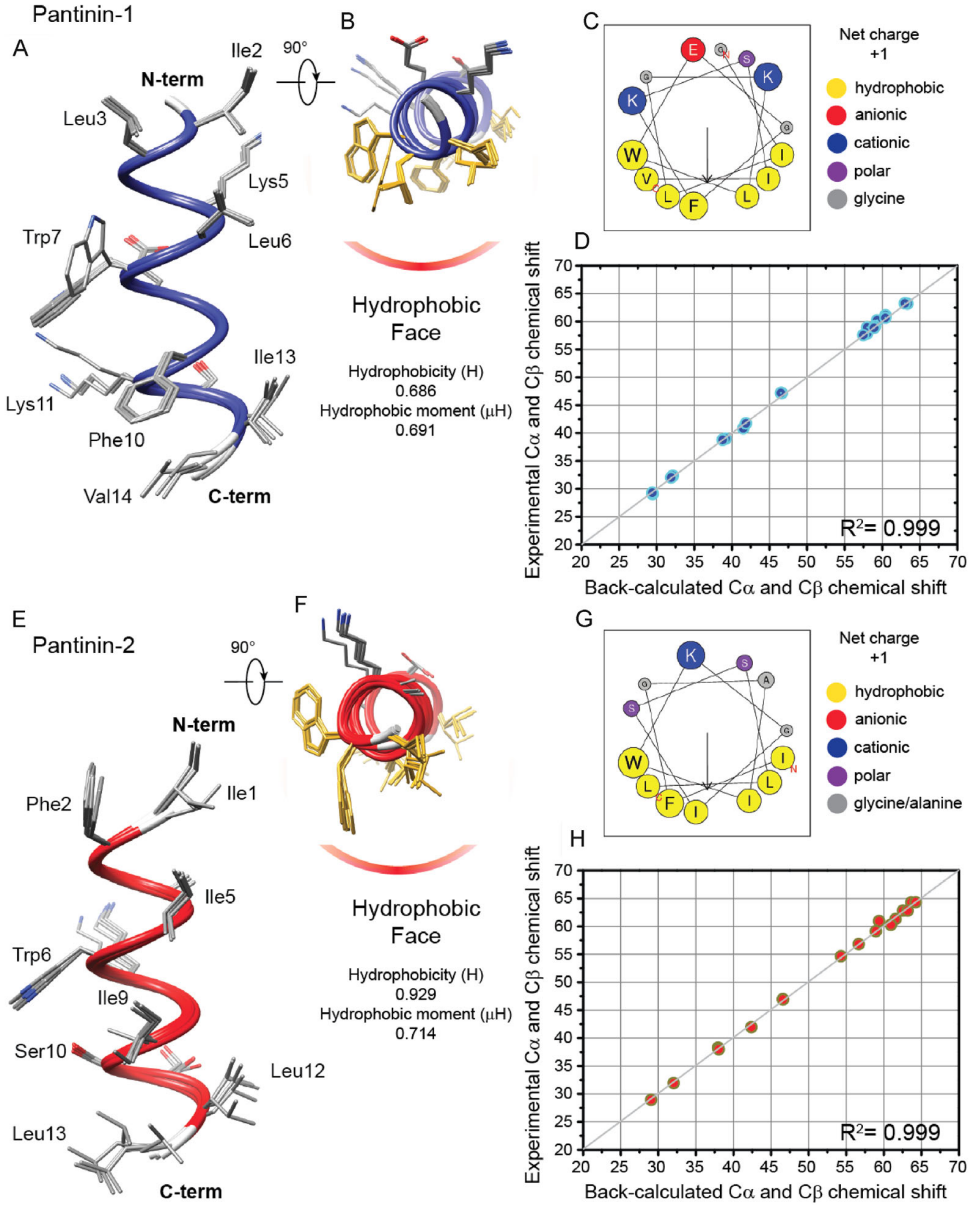
Prediction and validation of 3D structural models of pantinin‐derived peptides. A,B) Secondary structure (the residues are illustrated in blue) of pantinin‐1 predicted by PEP‐FOLD4 in two orientation (A,B) rotated by 90 around *x*‐axes. In panel B, the side chains of the residues composing the hydrophobic face, as indicated by the helix‐wheel diagram C), are depicted in yellow. D) Experimental versus back‐calculated C*α* and C*β* chemical shifts on the five‐best model structural ensemble. E,F) 3D structural models predicted for pantinin‐2 in which the residues in *α*‐helix are highlighted in red. In panel F, the side chain of the residues forming the hydrophobic face are shown in yellow, in according with the helix‐wheel diagram G). H) Correlation plot between experimental and back‐calculated C*α* and C*β* chemical shifts. This analysis was performed using the best five models predicted for pantinin‐2.

To better describe the structural peculiarities of pantinin‐1 and pantinin‐2, we predicted and validated their three‐dimensional (3D) structure using an integrated approach in which in‐silico techniques were implemented with experimental NMR structural data. In particular, 3D structural peptide models were calculated using PEP‐FOLD4^[^ ^51^ ^]^ method and then, as reported in the Materials and Methods section, the obtained structural ensembles were validated against the experimental NMR chemical shifts assigned for both peptides in the presence of 30% TFE (*v/v*) (**Figure** **9**A–H). The quality of predicted and selected models was determined by analyzing the dihedral angles phi (*φ*) and psi (*ψ*) through the Ramachandran plot indicating a remarkable quality for all conformational models with 100% residues in the most favored region (Figure SI2, Supporting Information). In the case of pantinin‐1, the predicted 3D structural ensemble, in agreement with the secondary chemical shifts, revealed that the peptide presents an amphipathic *α*‐helix, encompassing the segment of residues Ile2‐Ile13 (Figure 9A‐C), with a hydrophobicity (H) of 0.686 and a hydrophobic moment (μH) of 0.691. The hydrophobic face is composed by Ile2, Leu3, Leu6, Trp7, Phe10, Ile13, and Val14. For the pantinin‐2, in line with the secondary structure NMR data, the selected five best conformational models indicated that the peptide forms, in the region from Phe2‐Leu12, a continuous amphipathic helical conformation with a hydrophobic face (i.e., Ile1, Phe2, Ile5, Trp6, Ile9, Leu12 and Leu13), showing a hydrophobicity (H) of 0.929 and a hydrophobic moment (μH) of 0.714 (Figure 9E‐G).

To note, the greatest *α*‐helical peptide amphipathicity of pantainin‐2, most likely, is one of the main factors responsible for the observed greater antiviral potency. Finally, as mentioned above, we also validated the structural ensemble obtained for each of the two synthetic pantinin‐mimetic peptides by fitting the experimental C*α* and C*β* chemical shifts against the selected five models. Interestingly, in both cases, as reflected by R^2^ values, the experimental shifts are C*α* and C*β* in excellent agreement with the values predicted from the models, indicating that the predicted 3D peptide structures represent a faithful description of the peptide structural properties.

## 3 Conclusions

In the present study, we explored the structural properties and antiviral activity of pantinin‐1 and pantinin‐2, two synthetic peptides derived from scorpion venom. We provide the first evidence of their potent antiviral effects against two important veterinary alpha herpesviruses, BoHV‐1 and CpHV‐1. Our data demonstrate that both peptides exert their antiviral activity by directly interacting with the viral envelope, thereby interfering with the early phase of infection, including attachment and entry. Notably, pantinin‐2 exhibited greater antiviral potency, which may be attributed to its higher propensity to adopt *α*‐helical conformation and to the greater *α*‐helical peptide amphipathicity. Altogether, these findings highlight the potential of pantinin‐1 and pantinin‐2 as novel antiviral agents targeting enveloped viruses. Furthermore, the structural insights gained in this study offer a valuable foundation for the rational design of peptide analogs with enhanced stability and therapeutic efficacy.

## 4 Experimental Section

4.1

4.1.1

##### Peptide Synthesis and Characterization

Pantinin‐1 (single‐letter sequence: GILGKLWEGFKSIV‐NH2) and pantinin‐2 (IFGAIWKGISSLL‐NH2), amidated at the C‐terminus, were synthesized using the Fmoc‐solid phase peptide synthesis methodology. Oxime/DIC and HATU/sym‐collidine were used as coupling agents. The synthesis was carried out automatically using the SYRO I synthesizer (Biotage, Sweden AB), according to optimized protocols developed in our laboratory. Peptides purification and characterizations were performed as previously reported.^[^ ^34^ ^]^

##### Cells and Virus Culture

Madin‐Darby bovine kidney (MDBK, ATCC CCL‐22) cells were cultured in Dulbecco's Modified Eagle Medium (DMEM, Microgem, Naples, Italy) supplemented with 4.5 g L^−1^ glucose, 2 mM L‐glutamine, 100 IU mL^−1^ penicillin‐streptomycin solution, and 10% Fetal Bovine Serum (FBS) (Microgem). Cells were maintained in a humidified atmosphere with 5% CO_2_ at 37 °C. BoHV‐1 (Cooper strain) and CpHV‐1 (reference Swiss strain E/CH) were propagated and titrated using the MDBK cell line.

##### Cell Viability Assay

Cell viability was assessed using the methylthiazolyldiphenyl‐tetrazolium bromide (MTT) assay. In brief, MDBK cells (2 × 10^4^ cells/well) were seeded into 96‐well plates and incubated overnight (O/N) at 37 °C. The following day, cells were treated with serial dilutions of peptides (ranging from 3–100 μM) and incubated for 24 h at 37 °C. After treatment, 5 μL of MTT reagent (Sigma–Aldrich, St. Louis, MO, USA) was added to each well, followed by a 4 h incubation. Viable cells reduce the MTT reagent to insoluble formazan crystals via mitochondrial dehydrogenase activity.^[^ ^52^ ^]^ Cytotoxicity was calculated by measuring absorbance at 570 nm using a microplate reader.

##### Time of Addition Assays

To evaluate antiviral activity, MDBK cells (1.6 × 10^5^ cells/well) were seeded into 24‐well plates and treated with different concentrations of peptides (ranging from 3–50 µM) the following day. The peptides were administered at different stages relative to viral infection, as follows: (1) Cell pre‐treatment: to verify a possible interaction with cellular receptors, peptides were added to the cells for 1 h at 37 °C, followed by infection with the virus at a multiplicity of infection (MOI) of 0.01; (2) Virus pre‐treatment: to understand whether the action of peptides was directed at the viral particle, the virus (MOI = 0.1) was pre‐incubated with peptides for 1 h at 37 °C, after which the mixture was added to the cells and incubated for an additional hour at 37 °C; (3) Co‐treatment: to define if peptides could act during infection, therefore virus and peptides were added simultaneously to the cell monolayer and incubated together for 1 h; (4) Post‐treatment: to discriminate if the action of peptides was directed at late stages of the viral cycle, such as replication, cells were first infected with the virus at a MOI of 0.01 for 1 h, followed by the addition of peptides.

In all experimental conditions, after the 1 h adsorption period, the supernatant was removed and the cells were washed with citrate buffer. The monolayer was then overlaid with fresh culture medium containing 5% carboxymethylcellulose (CMC) (Sigma–Aldrich, Darmstadt, Germany). After 72 h of incubation, wells were emptied, washed with PBS, fixed with 4% formaldehyde for 20 min, and stained with 0.5% violet crystal.^[^ ^53^ ^]^ As a positive control (CTRL+), oreochromicin‐1, a peptide derived from the fish *Oreochromis niloticus,* well studied elsewhere, was used at a concentration of 25 µM.^[^ ^54^ ^]^ The negative control was represented by untreated infected cells. Viral inhibition was quantified by counting the number of plaques in peptide‐treated wells and comparing it to those in untreated infected controls in accordance with a following formula

(1) $$\%\;of\;\text{viral}\;\text{inhibition}=\left(1-\frac{\text{number}\;of\;\text{plaques}\;in\;\text{peptide}\;\text{treated}\;\text{infected}\;\text{cells}}{\text{number}\;of\;\text{plaques}\;in\;\text{untreated}\;\text{infected}\;\text{cells}\;}\right)$$

##### Temperature Shift Assays

Attachment assay: 1.6 × 10^5^ cells/well were seeded in a 24‐well plate and incubated O/N at 37 °C. Initially, the plate was pre‐cooled at 4 °C for 30 min. Then, cells were treated simultaneously with virus (MOI 0.01) and peptides for 1 h at 4 °C to allow virus attachment. After incubation, the supernatant was removed, and the cells were washed with citrate buffer. Plates were subsequently incubated at 37 °C for 72 h.

Entry assay: cells were plated and incubated as described above, then pre‐cooled at 4 °C for 30 min. Cell monolayer was infected with the viral suspension (MOI 0.01) and incubated at 4 °C to permit viral attachment. Following incubation, the supernatant was removed, and the cells were washed. Peptides were then added, and the plate was incubated at 37 °C for 1 h to allow viral entry. After treatment, the cells were covered with CMC and incubated at 37 °C for 72 h.^[^ ^55^ ^]^

##### Real‐Time PCR

Cells were seeded as described above, and infection was performed using the virus pre‐treatment approach. After 30 h post‐infection, total RNA was extracted using TRIzol reagent (Thermo Fisher Scientific, Waltham, MA, United States). RNA was reverse transcribed into cDNA using the 5× All‐In‐One RT MasterMix kit (Applied Biological Materials, Richmond, Canada). Quantitative real‐time PCR (qRT‐PCR) was then performed to assess the expression of the viral gene encoding glycoprotein B (gB). Cycle threshold (Ct) values were normalized to the housekeeping gene glyceraldehyde 3‐phosphate dehydrogenase (GAPDH), and relative mRNA expression levels were calculated using the 2^−^ΔΔCt method.^[^ ^56^ ^]^ Primer sequences used for qRT‐PCR are listed in **Table** **1**.

**Table 1:** Sequences of primers used for real‐time.

Gene	Forward sequence	Reverse sequence
gB	TGTGGACCTAAACCTCACGGT	GTAGTCGAGCAGACCCGTGTC
GAPDH	CCTTTCATTGAGCTCCAT	CGTACATGGGAGCGTC

##### Cell Morphology Analysis

To evaluate cell morphology during infection, acridine orange/propidium iodide (AO/PI) staining was performed. Briefly, viral particles and peptides were preincubated for 1 h before infection, and the resulting mixture was then applied to MDBK cells. After 48 h of incubation, cells were washed with PBS and stained with AO/PI for 15 min at 37 °C.^[^ ^57^ ^]^ Following staining, cells were washed again with PBS and examined using a ZOE Fluorescent Cell Imager (Bio‐Rad Laboratories, Hercules, CA, USA).

##### NMR Measurements and Chemical Shift Analysis

NMR experiments were acquired at 298K by using a Bruker AVIII HD 600 MHz spectrometer equipped with a triple‐resonance Prodigy N2 cryoprobe with *z*‐axis pulse field gradient.

NMR samples were prepared by dissolving the synthetic pantinin‐derived peptides in 200 μL of 5.0 mM sodium phosphate buffer, pH 7.4, and 10% ^2^H_2_O in a 3 mm tube. The final concentration was 0.4 mM. The chemical shift assignments of the three peptides were performed by following an optimized strategy consisting in the acquisition of the following spectra: i) Homonuclear bidimensional experiments: 2D [^1^H‐^1^H] TOtal Correlation SpectroscopY (TOCSY)^[^ ^58^ ^]^ and 2D [^1^H‐^1^H] Rotating frame Overhauser Effect SpectroscopY (ROESY)^[^ ^59^ ^]^ spectra were measured with 64 scans per t1 increment, a spectral width of 7211.54 Hz along both t1 and t2, 2048 × 300 complex points in t2 and t1, respectively, and 5.0 s relaxation delay. The water suppression was achieved using the Watergate pulse sequence with gradients using double echo. In the 2D [^1^H‐^1^H] TOCSY experiments, a DIPSI‐2 pulse sequence^[^ ^60^ ^]^ was used for isotropic mixing with a mixing time of 70 ms. 2D [^1^H‐^1^H ROESY] was acquired with a cw spinlock field strength of 4 kHz, a mixing time of 250 ms, and a 5.0 s relaxation delay. All 2D homo‐nuclear spectra were apodized with a square cosine window function and zero‐filled to a matrix of size 2048 × 2048 before Fourier transform and baseline correction; ii) Heteronuclear bidimensional experiments: 2D [^1^H‐^15^N] Hetero‐nuclear Single Quantum Coherence Spectroscopy (HSQC) experiment was acquired with 880 scans per t1 increment, a spectral width of 1581.26 Hz along t1 and 7211.54 Hz along t2, 2048 × 200 complex points in t2 and t1, respectively, and 1.0 s relaxation delay. The [^1^H‐^15^N] HSQC spectra were apodized with a square cosine window function and a zero filling to a matrix of size 4096 × 1024 before Fourier transform and baseline correction; 2D [^1^H‐^13^C] Hetero‐nuclear Single Quantum Coherence Spectroscopy (HSQC) spectrum was acquired with 800 scans per t1 increment, a spectral width of 1581.26 Hz along t1 and 7211.54 Hz along t2, 2048 × 200 complex points in t2 and t1, respectively, and 1.0 s relaxation delay. The [^1^H‐^13^C] HSQC constant time version ([^1^H‐^13^C] CT HSQC) was carried out with a heteronuclear coupling constant JXH = 145 Hz, constant time of 26.6 ms. The [^1^H‐^13^C] CT HSQC was apodized with a square cosine window function and zero‐filled to a matrix of size 4096x4096 before Fourier transform and baseline correction. ^1^H,^13^C, and ^15^N chemical shifts were calibrated indirectly by an external DSS reference. All 2D NMR spectra were processed by using NMRpipe^[^ ^61^ ^]^ and analyzed using SPARKY and CARA^[^ ^62^ ^]^ software. C*α*, H*α*, H_N,_ and C*β* secondary chemical shifts analysis was performed by using the random coil values defined by Kjaergaard et al.^[^ ^48,^, ^49^ ^]^ and Tamiola et al.^[^ ^47,^, ^50^ ^]^. The latter random coil values were used to estimate the secondary structure propensity.

##### Prediction and Validation of Three‐Dimensional Structures of Pantinin‐Derived Peptides

The structural ensembles of pantinin‐1 and pantinin‐2 peptides were obtained using the following protocol: i) peptide 3D structure models were predicted by PEP‐FOLD 4 server.^[^ ^51^ ^]^ In details, for each peptide the software calculated 100 models and then, the five lowest energy models were selected to generate the final ensemble; ii) After an accurate evaluation of the the quality and reliability of the predicted structural models by using the software PROCHECK^[^ ^63^ ^]^ the obtained structural ensembles were validated using experimental NMR data collected for both peptides upon addition of 30% TFE. In particular, the validation protocol consisted on the comparison between the experimental C*α* and C*β* chemical shifts and the values predicted from the structural ensemble generated in the previous step. The prediction of C*α* and C*β* chemical shifts was performed using PPM and PPM_ONE software.^[^ ^64^ ^]^ All 3D models selected for both peptides were analyzed and visualized using the CHIMERA software.^[^ ^65^ ^]^

The physicochemical features and helical wheel diagram of the two peptides were predicted using the HELIQUEST server.^[^ ^66^ ^]^

##### Statistical Analysis

All experiments were performed in triplicate, and the results are expressed as mean ± standard deviation (SD). Statistical analyses were conducted using two‐way ANOVA with Dunnett's multiple comparisons test. Graphs were generated with GraphPad Prism version 9.5.1 for macOS (GraphPad Software, San Diego, CA, USA; www.graphpad.com).

## Conflict of Interest

The authors declare no conflict of interest.

## Supporting information

Supplementary Material

## Data Availability

The data that supports the findings of the current investigation are available for the corresponding authors request.
